# Data-driven spatiotemporal assessment of the event-size distribution of the Groningen extraction-induced seismicity catalogue

**DOI:** 10.1038/s41598-022-14451-z

**Published:** 2022-06-16

**Authors:** A. G. Muntendam-Bos, N. Grobbe

**Affiliations:** 1 Department of Geoscience and Engineering, Delft University of Technology, 2600GA Delft, The Netherlands; 2 Dutch State Supervision of Mines, 2490AA The Hague, the Netherlands; 3 Hawai‘i Institute of Geophysics and Planetology, and Water Resources Research Center, University of Hawai‘I at Mānoa, Honolulu, HI 96822 USA

**Keywords:** Seismology, Natural hazards

## Abstract

For induced seismicity, the non-stationary, heterogeneous character of subsurface stress perturbations can be a source of spatiotemporal variations in the scaling of event sizes; one of the critical parameters controlling seismic hazard and risk. We demonstrate and test a systematic, statistical, penalized-likelihood approach to analysing both spatial and temporal variations in event size distributions. The methodology used is transferable to the risk analysis of any subsurface operation, especially for small earthquake catalogues. We explore the whole solution space and circumvent conventional, arbitrary choices that require a priori knowledge of these variations. We assess the effect of possible bias in the derivation, e.g., due to tapering of the earthquake-size distribution, correlation between the *b*-value and the magnitude of completeness and correlation between the *b*-value and the largest magnitude observed. We analyse the spatiotemporal variations in the earthquake-size distribution of the Groningen induced seismicity catalogue (December 1991–November 16, 2021). We find statistically significant spatial variations without any compelling, statistical evidence of a temporal variation. Furthermore, we find that the largest magnitudes observed are inconsistent with the sampling statistics of an unconstrained earthquake-size distribution. Current risk assessment models likely overestimate the probability of larger magnitude events (M ≥ 3.0) and thus the risk posed.

## Introduction

Anthropogenic activity that perturbs the stress in the subsurface can modulate seismicity (e.g.^1,2^). Over the last decades, the attention for the societal impact of human-induced earthquakes has been increasing. This increase is mostly due to a general increase in public awareness and concern^2,3^ as well as the regulatory response to the hazard and risk that these events may pose^2,4^. The regulatory response tends to focus on controlling the seismicity by modification or suspension of subsurface operations (e.g., for gas production, geothermal energy, CO_2_ capture, sequestration, and utilization, hydrogen storage)^5,6^, but can also encompass the application of traditional earthquake engineering to reduce building fragility and as such the associated risk^7^. A quantitative hazard and risk assessment must be capable of predicting changes in the seismic hazard and risk resulting from modifications to subsurface operations, combined with structural upgrading. The assessment forms the foundation for designing risk-based mitigation strategies that inform and guide operators, regulators, and governments in their respective roles and responsibilities^8–10^.

The scaling of earthquake sizes, the amount and temporal occurrence of events, and the maximum possible magnitude are among the critical parameters controlling seismic hazard and risk. Both the spatiotemporal variations in the scaling of event sizes and the maximum possible magnitude can impact the occurrence probability of larger magnitude events by orders of magnitude^11,12^. Spatial and temporal variations in the scaling of event sizes have been reported and were attributed a physical meaning (e.g.^11–15^). Hiemer and Kamer^14^ also showed that the performance of the Californian forecast models could be significantly improved when including large-scale spatial variations in the scaling of event sizes.

However, notwithstanding the vast literature on spatiotemporal variations, care should be taken in the evaluation and interpretation of these variations. Potential bias due to the evaluation of a (small) finite data set may lead to non-physical variations^16^. Spatiotemporal variations have been observed predominantly for small spatial regions^13–15,17–20^, and/or time windows^11–13^, while a constant event-size scaling holds well for larger areas^21^. In addition, the classical mapping technique for frequency-magnitude distributions^17,18^ depends heavily on external parameters for which the parameter-value choices require a priori knowledge of the spatial or temporal event-size distribution that one wishes to resolve in the first place^19^. As such, there is a need for methodologies for spatiotemporal analyses that circumvent the arbitrary choices in these mapping parameters. Kamer and Hiemer^20^ introduced a parameter-free method based on optimal partitioning using Voronoi tessellation, thereby exploring the whole solution space. The method used a penalized-likelihood approach and the wisdom of the crowd philosophy. The authors showed that the circumvention of arbitrary parameter choices improves the mapping of spatial variations. In theory, the methodology can be extended to also include the temporal dimension, but this has, to our knowledge, not been done so far.

**Figure 1:**
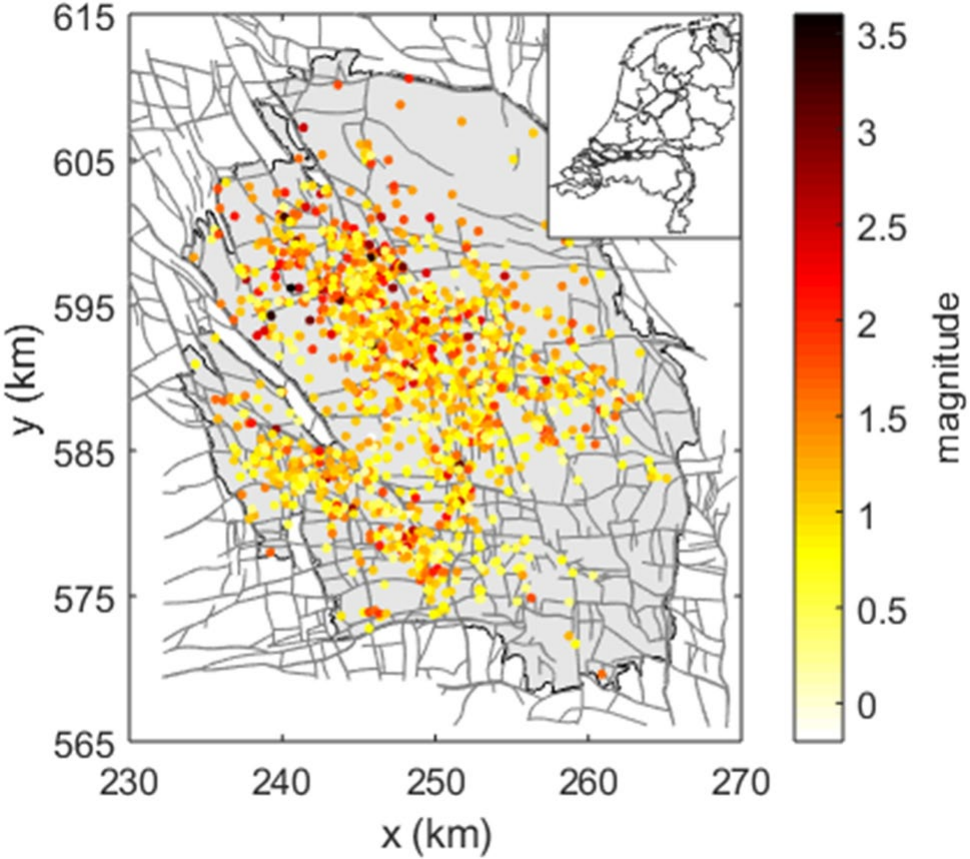
Overview of the epicentre locations of the Groningen induced seismicity for the period December 1991–November 16, 2021. The colours indicate the different local magnitudes of the events.

The Groningen gas field, located in the north-east of the Netherlands, is the largest gas field in Europe and the tenth largest in the world (Fig. 1). In recent years, it was one of the most studied fields in terms of induced seismicity (e.g.^22–27^). At the same time, a lot of open questions and concerns remain. The field has been associated with induced seismicity since December 5, 1991, with the largest event to date being the August 16, 2012 Huizinge event with a local magnitude ${M}_{l}3.6$. Its societal impact was tremendous, causing damage to buildings and health concerns for the local population^28^.

Probabilistic seismic hazard and risk assessments help inform and guide government policy and operational strategies, and provide the regulator with quantitative hazard and risk insights. To this end, it is absolutely vital that hazard and risk calculations are accurate, precise, and reflect the state-of-the-art of science and technology. This includes the need for a systematic and accurate evaluation and interpretation of spatiotemporal variations in event size distribution. Especially, given the significant impact of these variations on the occurrence probability of larger magnitude events. The need for such a systematic, unbiased spatiotemporal analysis procedure does not only apply to the Groningen gas field, but is transferable to, and of the utmost relevance for, accurate risk analysis of any subsurface operation potentially causing induced seismicity. In particular, when the corresponding earthquake catalogues are relatively small. This includes, but is certainly not limited to, geothermal energy, CO_2_ capture, sequestration and utilization, and hydrogen storage operations, all highly relevant within the context of the global energy transition.

For the Groningen gas field, probabilistic seismic hazard assessments (PSHA) have been performed by the KNMI (Royal Dutch Meteorological Survey) since 2006^22,29^. In these PSHA’s, KNMI has implemented a seismic source model with a zonation based on event density, known faults and information on compaction. In the model, the scaling of event sizes is zone dependent, but stationary in time and estimated from the recorded seismicity within each zone. In contrast, Bourne et al.^24^ introduced a stress-dependent scaling of event sizes in the operator’s PSHA. This stress dependence ought to be consistent with a spatiotemporal variation of event sizes, as stresses increase with time due to the gas production. In their model, stresses were fault offset and compaction dependent^30,31^. Recently, Bourne and Oates^32^ concluded that the stress dependence is not located in the scaling of event sizes, but rather in an exponential taper reducing the probability of larger magnitude events. Despite the fact that accounting for these variations in the earthquake-size distribution impacts the seismic hazard and risk significantly, a rigorous, data-driven assessment of the validity of the spatiotemporal variations is still unresolved.

In this paper we demonstrate and test a systematic approach to analysing both spatial and temporal variations in event size distributions. We assess whether spatiotemporal variations in the earthquake-size distribution exist in the Groningen induced seismicity catalogue. First, we analyse possible spatial variations in the *b*-value. To circumvent the arbitrary choices in external mapping parameters, we adopt the method introduced by Kamer and Hiemer^20^. We modify and expand the methodology to include the temporal dimension in the analysis. Throughout our analysis we will systematically explore the effect of possible sources of bias on the derived spatiotemporal variations. Our methodology is transferable and applicable to any induced-seismicity setting. It is particularly relevant for relatively small earthquake catalogues, and provides a rigorous and unbiased approach to untangling potential spatiotemporal variations and their physical meaning. At the same time, our findings could have consequences for future hazard and risk calculations for the Groningen gas field, and help inform important decision-making processes.

## Data

Natural gas has been produced from the Groningen gas field since 1963. At present, about 70% of the estimated 2800 × 10^9^ m^3^ initial gas in place has been produced, dropping the initial mean pore pressure by about 25 MPa. The field is located in the sandstones of the Rotliegend formation, which is overlain by a thick layer of Zechstein halite and anhydrite salt deposits^25^. The reservoir is highly faulted with over 1100 mapped, steeply dipping normal (extensional) faults (Fig. 1). We refer the reader to^25^ for more detailed information on the geology of the Groningen gas field.

The earthquakes in the Groningen gas field are induced by gas extraction at a depth of approximately 3 km^33^ and have relatively small magnitudes (${M}_{l}\le 3.6$) (Fig. 1^22,33^). A local geophone network with a detection threshold of local magnitude ${M}_{l}=1.5$ was installed in 1995^22^. In 2015, the geophone network was significantly extended to increase the detection of small magnitude earthquakes^22^. In total, 1396 events were detected between December 1991 and November 16, 2021, ranging from local magnitudes ${M}_{l}=$ − 0.5 to 3.6, with fourteen events ${M}_{l}\ge 3.0$ (e.g.^22^). In this paper, we have used the catalogue reported by the KNMI (www.knmi.nl), from which we have selected all events within the outline of the Groningen gas field (Fig. 1). Note that in this catalogue a hypocentre depth of 3 km has been assumed a priori^22^.

## Method

### Earthquake-size distribution

The relation between the cumulative number of earthquakes (*N*) and magnitude (*M*) follows a power-law distribution expressed as ${\mathrm{log}}_{10}N=a-b(M-{M}_{c})$^34^, where ${M}_{c}$ is the magnitude of completeness and *a* and *b* (so-called *b*-value) are constants that describe the productivity and the relative size distribution, respectively.

In the analysis presented in this paper, the *b*-value was determined with the maximum likelihood method^35,36^, following Kagan^21^. In order to avoid bias in the *b*-value estimates^16^, we implemented a correction for magnitude binning^16^ and small sample sizes^37^.

The (regional) magnitude of completeness was calculated with the maximum curvature method (MCM^38^). The advantage of the MCM is that results can be obtained fast and reliably, even for small sample sizes. On the other hand, the method tends to underestimate ${M}_{c}$, especially for gradually-curved frequency-magnitude distributions. This disadvantage can be overcome by using a correction factor (${M}_{c}= {M}_{c}\left(MCM\right)+ \Delta {M}_{c}$) in combination with the bootstrap approach^38^. After careful assessment of the (regional) MCM results, while increasing the correction factor ($\Delta {M}_{c}$), an initial correction factor of $\Delta {M}_{c}=0.2$ was adopted.

### Penalized likelihood-based method

The classical spatial *b*-value mapping technique^17,18^ depends heavily on external parameters for which the parameter-value choices require a priori knowledge of the spatial event-size distribution that one wishes to resolve in the first place^19^. The penalized likelihood-based method of Kamer and Hiemer^20^ addresses these limitations. This parameter-free method is based on optimal partitioning using Voronoi tessellation, thereby exploring the whole solution space. The method uses a penalized-likelihood approach and the wisdom of the crowd philosophy^20^.

Voronoi tessellation partitions the space using a set of points (nodes) and assigns each node its nearest neighbourhood region. By random perturbation of the nodes, arbitrarily shaped and sized regions are obtained. The approach thus allows for a flexible, non-overlapping partitioning in space. The overall log-likelihood of each random tessellation can be computed by estimating the *b*-value in all Voronoi regions, computing the log-likelihood of each region, and subsequently summing the log likelihoods of the Voronoi regions^20^.

The overall log-likelihoods were subsequently penalized based on the number of free parameters by using the Bayesian Information Criterion (BIC)^39^, given by $BIC= -\mathrm{log}\widehat{L}+\frac{k}{2}\mathrm{log}N$, where $\widehat{L}$ is the overall likelihood, $k$ represents the number of free parameters and $N$ denotes the number of data points. Finally, all models were ranked by their BIC and the median BIC weighted ensemble model was calculated using the solutions outperforming a chosen null hypothesis, with a maximum of the 1000 best solutions. Thus, models of different complexity but with similar BIC had equal influence on the ensemble inference, which is a manifestation of the wisdom of the crowd philosophy.

**Figure 2:**
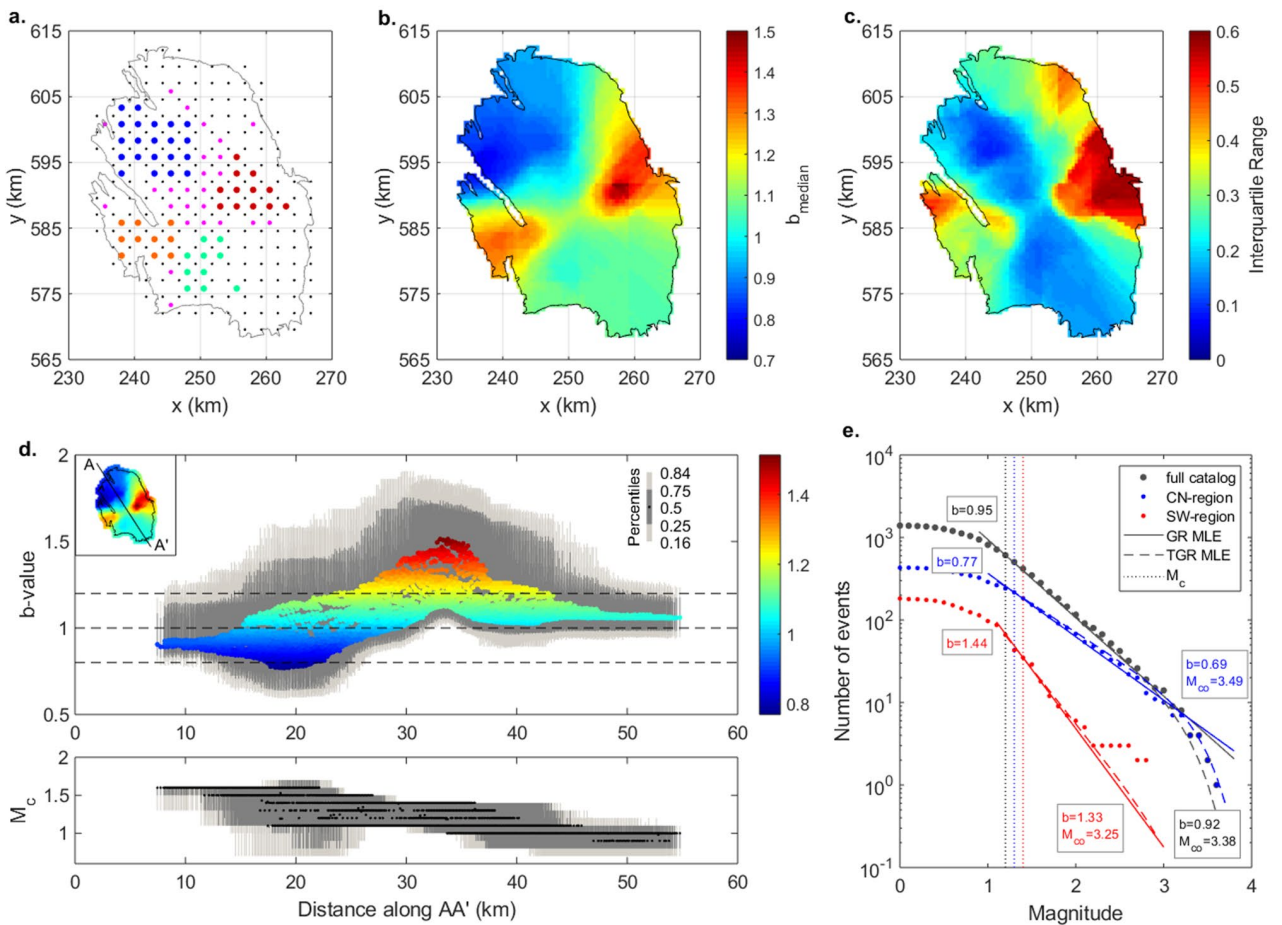
Results of the penalized likelihood-based analysis of possible spatial variations of the b-value within the Groningen catalogue. (**a**) Overview of the discretization of the spatial domain by a 2.5 × 2.5 km grid (all dots). Only cells containing more than one event are considered a potential node location (all coloured dots). Blue node locations (blue dots) denote the north-western (NW) region; Orange node locations (orange dots) denote the south-western (SW) region; Red node locations (red dots) denote the central-eastern (CE) region; Cyan node locations (cyan dots) denote the central-southern (CS) region. (**b**) Ensemble median b-value based on the best 1000 solutions. (**c**). The corresponding ensemble interquartile range of the best 1000 solutions. (**d**) Median *b*-values from (**b**). and median magnitude of completeness (${M}_{c}$) as a function of the distance along the cross-section A-A’. Dark and light grey bars correspond to percentiles of (0.25–0.75) and (0.16–0.84), respectively. (**e**) Cumulative earthquake-size distribution for the full Groningen catalogue (black), NW-region (blue) and SW-region (red), with their corresponding maximum likelihood estimate (MLE) fits for the non-tapered (solid line) and tapered (dotted line) model distributions. Respective *b*-values and corner magnitudes (${M}_{co}$) are indicated on the panel.

We discretized the Voronoi selection space, in accordance with the event location uncertainties, in 2.5 × 2.5 km cells (Fig. 2a). Only cells with at least two events ${M}_{l}\ge {M}_{c}$ were considered a potential Voronoi node location (red dots in Fig. 2a). Each set of nodes partitioned the assessment space in anisotropic regions of various shapes and sizes. Increasing the total number of nodes allowed for the exploration of smaller scale variations.

The assessment of a single Voronoi cell, i.e., having no spatial variation, was regarded as the null hypothesis. Subsequently, the number of nodes considered in an analysis was increased from 2 to 50, performing 2000 random tessellations at each step. For each Voronoi cell in each tessellation, both the ${M}_{c}$ and the *b*-value were estimated. Moreover, a consistency check on, and if necessary a correction of, the ${M}_{c}$ correction factor was performed. If no ${M}_{c}$ could be derived due to too small a sample size the ${M}_{c}$ of the null-hypothesis was adopted (Table 1). This occurred in only 2% of the estimations and as such did not have a substantial impact on the final results.

**Table 1:** Overview of the parameter estimates of the (tapered) earthquake-size distributions and the Akaike Information Criterion corrected for small sample sizes (AICc) test statistics.

Region	$N$	${M}_{max}^{obs}$	${M}_{c}$	Non-tapered		Tapered			
				$b$	$AICc$	$b$	${M}_{co}$	$AICc$	$\nu$
Full	610	3.6	1.2	0.95 ± 0.04	34,204	0.92 ± 0.04	3.38 ± 1.23	34,203	0.59
NW	216	3.6	1.3	0.77 ± 0.08	12,455	0.69 ± 0.08	3.49 ± 1.37	12,455	0.64
SW	35	2.8	1.4	1.44 ± 0.31	1952.4	1.33 ± 0.33	3.25 ± 1.14	1954.7	4.67
CE	62	2.5	1.2	1.53 ± 0.31	3361.6	1.41 ± 0.33	2.25 ± 0.93	3367.4	0.86
CS	85	3.1	1.0	0.96 ± 0.13	4632.7	0.87 ± 0.16	2.65 ± 1.39	4635.5	0.73
Full < 2014	319	3.6	1.3	1.00 ± 0.08	18,065	0.97 ± 0.09	3.57 ± 1.42	18,066	1.04
Full > = 2014	366	3.4	0.9	0.88 ± 0.06	19,957	0.84 ± 0.06	3.32 ± 1.01	19,957	0.72

N indicates the number of events with ${M}_{l}\ge {M}_{c}$, ${M}_{max}^{obs}$ is the magnitude of the largest event in the dataset, $b$ is the *b*-value estimate with its 1 − σ uncertainty, ${M}_{co}$ is the estimated corner magnitude with its 1 − σ uncertainty, and ν is the coefficient of variation. Regions (see Fig. 2a): *NW* northwest, *SW* southwest; *CE* central eastern region, *CS* central southern region.

To derive temporal *b*-value variations devoid of choices that require a priori knowledge, we have adapted the spatial approach; a non-straightforward advance. We discretized the temporal selection space in 5-year cells, offset with respect to the analysis times by 0.33 years. This offset ensured that cell partitions did not align with the temporal analysis locations. Only cells or combinations of neighbouring cells with at least 20 events ${M}_{l}\ge {M}_{c}$ were considered a potential node location. We increased the number of minimum events relative to the spatial assessment, because the discretization was only one-dimensional (in time). This one-dimensionality of our temporal problem increased the probability that neighbouring cells were selected, and the minimum number of events would form the basis of the assessment of the cell. A very low number of minimum events would then introduce an extreme bias due to very small sample sizes.

Similarly to the spatial analysis, we again regarded the assessment of a single cell, i.e. having no temporal variation, as the null hypothesis. The number of nodes $$\left({n}_{n}\right)$$ considered was increased from 2 to $$({n}_{n}^{t}-1)$$, where $${n}_{n}^{t}$$ was the total number of potential node locations. By limiting the minimum number of events to 20, the temporal tessellation resulted in a limited number of potential node locations. Hence, it was important to avoid superfluous repetition of particular tessellations, as these would bias our results. The maximum number of unique random tessellations is limited by both the number of nodes considered $$\left({n}_{n}\right)$$ and the total number of potential node locations $$({n}_{n}^{t})$$. The number of unique random tessellations of $${n}_{n}^{t}$$ nodes when considering $${n}_{n}$$ nodes ($${}_{{n}_{n}^{t}}{}{T}_{{n}_{n}})$$ can be computed by $${}_{{n}_{n}^{t}}{}{T}_{{n}_{n}}={n}_{n}^{t}!/{n}_{n}!\left({n}_{n}^{t}-{n}_{n}\right)!$$. In our analysis, we wanted to ensure all unique tessellations were considered, while avoiding superfluous repetitions; therefore, we restricted the total number of random tessellations at each step to $$2{}_{{n}_{n}^{t}}{}{T}_{{n}_{n}}$$ with a maximum of 2000.

## Results

### Spatial variations in earthquake-size distribution

We first investigated possible spatial variations in the *b*-values in the Groningen gas field. The median *b*-values derived with the penalized likelihood-based method are shown in Fig. 2b and range from 0.77 to 1.52. We observe a very systematic division between low *b-*values in the north-northwest of the field (i.e., a relative abundance of larger earthquakes) and higher *b-*values in the west and east (i.e., a relative abundance of smaller earthquakes; Fig. 2d). The high *b-*value region in the east is less well defined and associated with a large interquartile range (Fig. 2c).

To complement the assessment of the statistical significance of this spatial pattern, we derived regions of comparable *b*-values from our spatial solution. The nodes assigned to each region are indicated in Fig. 2a by the different node colours. For each region, we computed the average regional *b-*value and ${M}_{c}$ based on all enclosed events (Table 1; Fig. 2e). The *b-*values obtained for the southwest (SW) and central-eastern (CE) regions are slightly larger than determined in the Voronoi analysis. This is consistent with observations of Kamer and Hiemer^20^, that *b-*values in a high *b-*value area may be underestimated by the Voronoi approach. We use the two sample, left-tailed t-test or Welch’s test^40^ to assess whether the derived regional *b-*value distributions are in fact samples of the same, larger distribution (e.g., the single distribution for the full Groningen catalogue):

$$t= \frac{\widehat{{b}_{1}}-\widehat{{b}_{2}}}{\sqrt{\frac{{s}_{1}^{2}}{{n}_{1}}+\frac{{s}_{2}^{2}}{{n}_{2}}}},$$

where $\widehat{{b}_{1}}$ and $\widehat{{b}_{2}}$ are the derived *b*-value estimates, *s*_*1*_ and *s*_*2*_ are the standard deviations of the two estimates, and *n*_*1*_ and *n*_*2*_ are the sample sizes. We find that the probability that the regional *b-*values of the northwest (NW), SW and CE regions are samples of a single *b-*value distribution, is less than 5%. This further confirms that the obtained spatial distribution of the *b*-value is statistically significant at the 95% confidence level.

### Bias introduced by tapering of the earthquake-size distribution

The limited capability to accumulate seismic energy in one specific region or on a single fault requires the earthquake-size distribution to decay stronger above a particular magnitude called the corner magnitude ${M}_{co}$ (Fig. 2e). This tapering of the distribution may introduce a bias in the estimation of the *b*-value^16,32,41,42^. As the Groningen catalogue is limited in magnitude range with ${M}_{c}$ ranging from 0.8 to 1.5^22^ and a maximum observed magnitude of ${M}_{l}$=3.6, our derived regional, spatial *b*-value estimates may be influenced. Here, we explored this possible bias by jointly deriving the maximum likelihood estimates of the *b*-value and ${M}_{co}$ for the tapered distribution (Table 1^21^).

We find that in all our analyses, the *b*-value of the tapered estimation is systematically lower than the *b*-value derived in the non-tapered estimation. This is directly related to the significant positive correlation between the estimation of the corner magnitude and the *b*-value: an increase in the corner magnitude estimate is compensated by an increase in the *b*-value estimate^21^. Kagan^21^ further showed that the correlation coefficient increases if the difference between ${M}_{co}$ and ${M}_{c}$ is small.

We used the corrected Akaike Information Criterion (AICc^43^) to assess and compare the fit of both the tapered and non-tapered models to the data (Table 1). In most regions, the relative probability is comparable or the non-tapered model is favoured. Only for the full catalogue the AICc of the tapered model is slightly lower than the AICc of the non-tapered model. Another estimate of the probability of the non-tapered model rejection can be obtained by analysing the coefficient of variation ($\nu$ in Table 1^21^). If the coefficient of variation is close to, or larger than, 0.5, the non-tapered model (${M}_{co}\to \infty$) is within the 97.5% confidence interval. Based on this test, the non-tapered model cannot be rejected in any of the analyses. However, the coefficient of variation for the full (0.59) and NW-region (0.64) catalogues are just outside the 97.5% confidence interval. We conclude that our relatively small earthquake catalogues yield little to no statistical information on the presence of a taper. Possible bias due to tapering of the earthquake-size distribution on the non-tapered *b*-value estimates can be regarded negligible. However, our results raise the question whether the earthquake magnitudes are as large as statistically expected? We will investigate this further after the assessment of possible temporal variations in the earthquake-size distribution.

### Temporal variations in earthquake-size distribution

Following the analysis of the spatial variations, we also assessed the possible presence of temporal variations in the earthquake-size distribution. Initially, we attempted to extend the spatial analysis by adding the temporal dimension. However, due to the heterogeneous spatiotemporal development of the Groningen seismicity, this did not render meaningful results. Therefore, we have adapted the penalized likelihood-based method for the temporal domain (see “Method” section) and applied this to the full Groningen catalogue.

**Figure 3:**
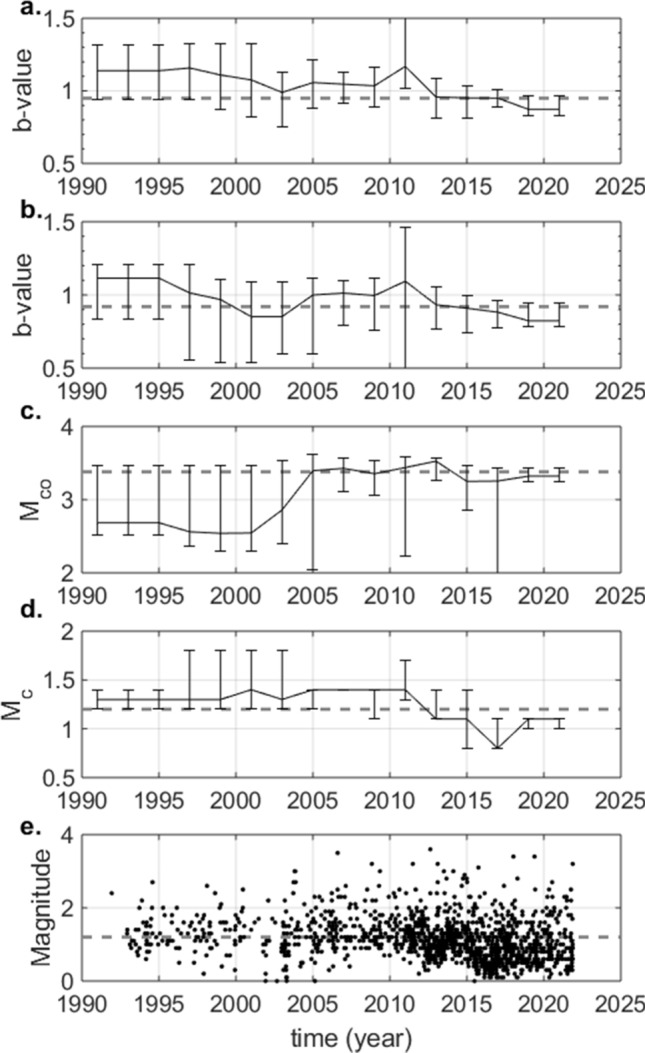
Result of the penalized likelihood-based analysis of possible temporal variations of the *b*-value in Groningen. (**a**) Temporal development of the *b*-value for the non-tapered solution. (**b**) Temporal development of the *b*-value of the tapered solution. (**c**) Temporal development of the corner magnitude of the tapered solution. (**d**) Temporal development of the magnitude of completeness. In (**a**–**d**) the error-bars denote the 5th to 95th percentiles range and the dashed line shows the value derived for the null-hypothesis of no temporal variations. (**d**) Magnitudes of the events in the catalogue plotted versus time.

The results of our assessment for the non-tapered solution are shown in Fig. 3a. The results for the *b*-value and corner magnitude of the tapered solution are shown in Fig. 3b,c, respectively. In both solutions, we obtained a slightly decreasing *b*-value with time, which seems insignificant at the 90% confidence level. At the same time, the tapered solution shows an indication of an increasing corner magnitude with time. However, this is also insignificant at the 90% confidence level.

To complement the assessment, we split the dataset into two catalogues of approximately equal size. The first catalogue contained only events prior to 2014, the second only events from 2014 to 2022. For each dataset we derived the tapered and non-tapered solutions (Table 1). We find, by comparing the AICc’s, that the events prior to 2014 (Table 1) may be better described by a non-tapered distribution, with a slightly larger *b*-value of 1.00 ± 0.08. The second half of the dataset (between 2014 and 2022) are described equally well by the tapered and non-tapered distributions. The two sample, left-tailed t-test confirms that the *b*-value distributions for the two periods may be samples of the same, larger distribution for the full dataset (the difference is insignificant at the 90% confidence level).

### Bias due to correlation between the b-value and the largest magnitude

Even though the maximum likelihood estimation takes into account only the small magnitudes in the catalogue and not the largest, a correlation between the *b*-value and the largest magnitude of the dataset may remain, especially for relatively small datasets. This is directly related to the fact that the mean of an exponential distribution is sensitive to outliers. As a consequence, the *b*-value decreases as the largest magnitude in the dataset increases^16^.

**Figure 4:**
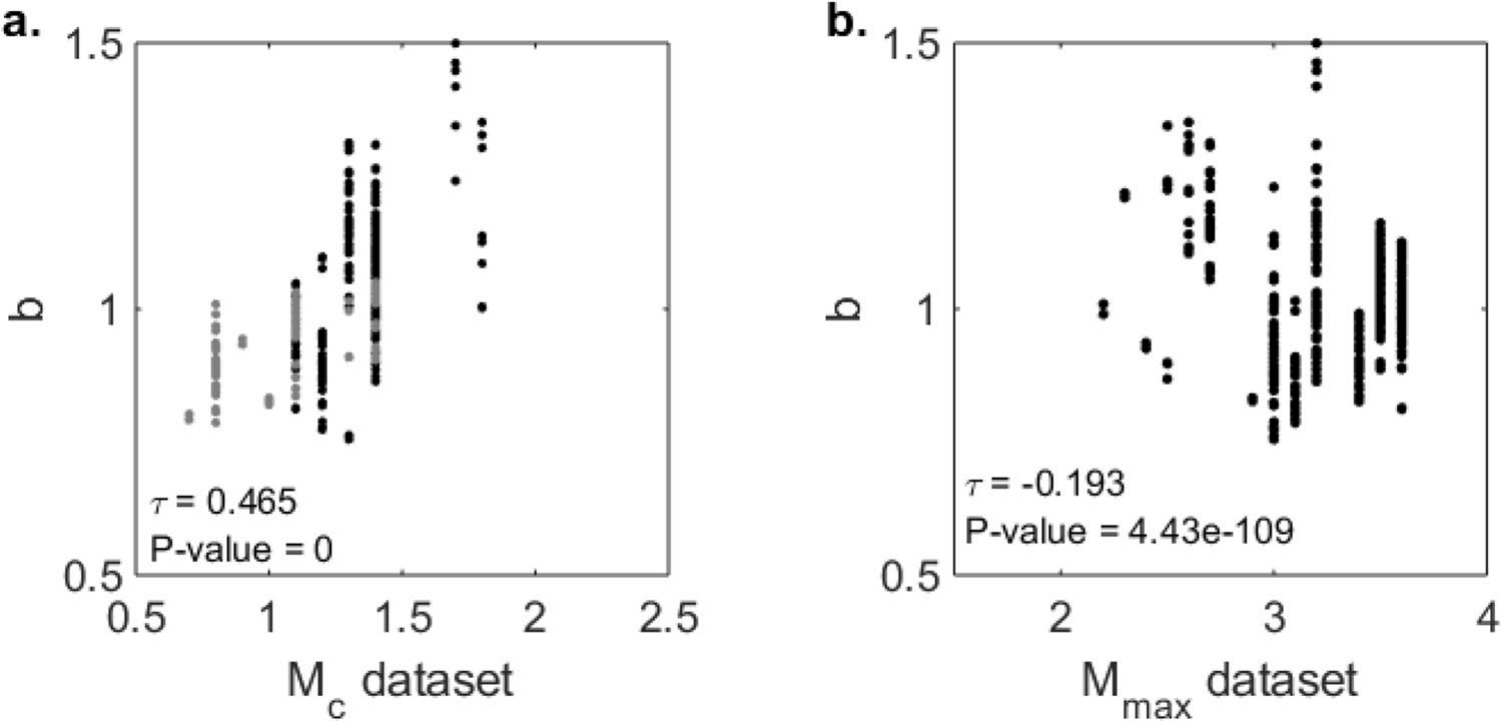
Plots of the *b*-values derived in the solutions used to compute the temporal median *b*-values as a function of magnitude of completeness **(a**) and largest observed magnitude in the dataset analysed (**b**). (**a**) *b*-value as a function of magnitude of completeness for the temporal analysis. The grey dots correspond to the solutions for the temporal nodes after 1-1-2014. (**b**) *b*-value as a function of the largest observed magnitude in the dataset for the temporal analysis. In each plot we report the correlation coefficient τ and the P-value obtained by the Kendall-Tau test for the null hypothesis of no correlation.

Figure 4b shows a plot of the *b*-values as a function of the largest magnitude in the dataset analysed. The results of the Kendall-Tau significance test^44^ for the correlation are shown. A very small, but significant negative correlation between the *b*-value and the largest magnitude in the dataset was obtained. This suggests that we observe a slightly lower *b*-value when the dataset contains at least one stronger event. However, the timing of the observed decrease (between 2010 and 2015) does not correlate with the observed increase in larger magnitude events (in 2003 and 2006; Fig. 3e). We conclude that the observed apparent decrease in *b*-value is not related to the sensitivity of the maximum likelihood estimate due to the onset of ${M}_{l}\ge 3.0$ events in 2003.

### Bias due to correlation between the b-value and the magnitude of completeness

Incompleteness of the earthquake catalogue at low magnitudes can introduce a significant bias on the estimation of the *b*-value: underestimating ${M}_{c}$ leads to an underestimation of the *b*-value^16^. Figure 4a shows the *b*-values derived in the solutions used to compute the temporal values, shown in Fig. 3a, as a function of the magnitude of completeness in the datasets analysed. The results of the Kendall-Tau test^44^ are again shown (Fig. 4a). A significant positive correlation between the *b*-value and the magnitude of completeness was obtained. Closer examination (Fig. 3d) shows that the period of low *b*-values and low ${M}_{c}$ occurs predominantly after 2014. The decrease in ${M}_{c}$ is directly related to a significant extension of the seismic monitoring network and is consistent with previous assessments for this time period^22^. In addition, Fig. 4a shows that the correlation between the *b*-values and ${M}_{c}$ for the period as of 2014 (grey dots) is much less pronounced. Therefore, we conclude that the observed correlation is not the result of a bias due to underestimation of ${M}_{c}$.

### Are the earthquake magnitudes as large as statistically expected?

**Figure 5:**
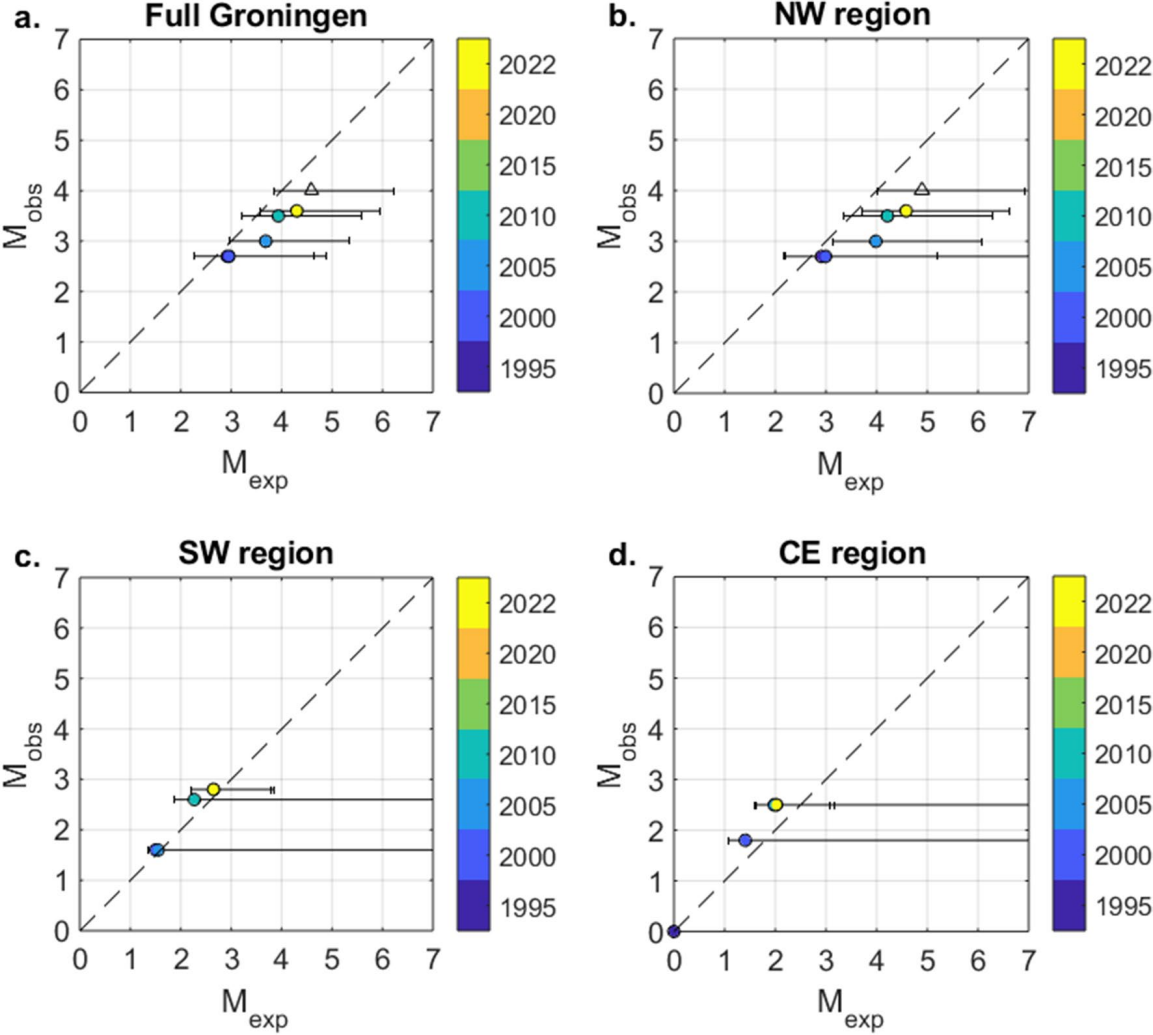
Plot of the temporal development of the largest magnitude observed as a function of expected maximum magnitude. The horizontal error bars show the 90% confidence ranges for the expected maximum magnitude. On the diagonal, dotted line the largest magnitude observed and expected would be identical. If the 90% confidence range does not overlap with the diagonal line, the probability that the largest magnitude observed scales logarithmically with the number of earthquakes is less than 5%. In all analyses, the onset of the time interval is 1-1-1990. The colour bar indicates the year at the end of the time interval assessed: 1-1-year. The data in the most recent interval extends to 16-11-2021, i.e., the end of the catalogue used. Note that in all panels some of the time intervals are not visible as the observed and expected maximum magnitudes are (almost) identical to the following time intervals and for the CE-region no events were observed prior to 1-1-1995 (hence the dot at (0,0)). (**a**) Full Groningen catalogue. (**b**) Sub-catalogue of the NW-region. (**c**) Sub-catalogue of the SW-region. (**d**) Sub-catalogue of the CE-region. The open triangles indicate the expected maximum magnitude for the (regional) databases of the NW-region and full catalogue with a hypothetical event of ${M}_{l}$ 4 on 1-7-2022.

Our results raise the question whether there exists an intrinsic limit on the maximum size of the induced earthquakes in Groningen. Following Van der Elst et al.^45^, we computed the statistically expected maximum magnitude range for each of the sub-catalogues with time. In Fig. 5, the largest magnitude observed is plotted as a function of the largest magnitude expected. For the full Groningen catalogue, we find that the largest magnitude observed is consistently low compared to what could be expected, but just within the 90% confidence range. For the NW-region, we find that, as of 2005, the observed maximum magnitude is significantly smaller than statistically expected and falls outside the 90% confidence range of the expected distribution. Prior to 2005, and in the SW and CE regions, the observed magnitudes are as large as can be statistically expected.

## Discussion

Our results suggest that there is clear, statistically significant evidence for spatial variations in the earthquake-size distribution of the induced seismicity sequence of Groningen. We obtained low *b-*values of ~ 0.8 in the northwest (NW) of the field and high *b*-values of ~ 1.5 in the south-western and eastern parts of the field (Table 1). We found no compelling, statistical evidence of a temporal variation of the *b*-values in the Groningen gas field.

Further, our results clearly showed that our relatively small earthquake catalogues yield little to no statistical information on the presence of a taper. In most analyses, the relative probability of the two models was comparable, or the non-tapered model was favoured. Only for the full catalogue, the AICc of the tapered model was slightly lower than the AICc of the non-tapered model with a coefficient of variation of 0.59, just outside the 97.5% confidence interval. Also for the NW-region, the coefficient of variation of 0.64 was close to this interval. This hints towards an area-characteristic corner or maximum possible magnitude.

The Groningen dataset contains no ${M}_{l}\ge 3.0$ events prior to 2003. However, this is not remarkable. Based on the sampling statistics of the earthquake-size distribution, the largest observed earthquake up to 2005 in all regions is consistent with the largest magnitude expected (Fig. 5). Figure 5 also shows that in all regions, the largest magnitude observed has increased with time. This is inconsistent with the case where the earthquake size is determined by natural tectonics, as in that case each earthquake would have the same probability of becoming the largest^45^. In fact, production from the Groningen gas field occurred for 20–25 years without any seismicity being recorded. Since the onset of seismicity, the increases in both the number of events and the largest magnitude observed are consistent with a progressive destabilization of the pre-existing faults under increasing Coulomb stress due to reservoir depletion^46^.

We found statistical evidence of a stronger decay of the occurrence probability for the larger magnitude events as the catalogue increases (Fig. 5). The observed largest magnitudes after 2005 were significantly lower and at the edge of the 90% confidence range of the expected maximum magnitude. This means that the probability that the largest magnitude observed scales logarithmically with the number of earthquakes, is approximately 5%. Figure 5 also shows that the occurrence of a larger magnitude event later in time can mean that the largest magnitude observed again approximates or falls just within the 90% confidence range of the maximum magnitude expected (e.g., due to the ${M}_{l}3.5$ event in 2006). We therefore found it important to investigate if a future event, significantly larger than the current maximum observed, would result in a similar reconciliation. In both the full Groningen and NW-region, we extended the sub-catalogue with an additional fictitious event (${M}_{l}4.0$ ) to occur on the first of July 2022. The expected maximum magnitudes are given by the open triangles in Fig. 5. In the NW-region, the fictitious observed ${M}_{l}4.0$ still falls outside the 90% confidence range of the corresponding expected maximum magnitude. For the full Groningen catalogue, the fictitious ${M}_{l}4.0$ event is still on the low side, but would fall just within the 90% confidence range of the expected maximum magnitude. This difference can be mostly explained by the significantly larger *b*-value obtained for the full catalogue compared to the NW region, which lowers the probability of LME’s (Table 1). This analysis further confirms that, after taking into consideration the statistically significant spatial variations of the *b*-value, only the data from the NW-region contains some statistical information on the position of a possible corner magnitude or maximum possible magnitude, which could provide an upper bound to the earthquake-size distribution. Our analysis again shows how notoriously difficult it is to constrain a magnitude bound or taper from observed seismicity alone^47,48^.

In 2016, a conditional distribution for the maximum possible magnitude (${M}_{max}$), based on statistical and model considerations, was published for Groningen^49^. The distribution extends from ${M}_{l}3.8$ to ${M}_{l}7.2$, with a weighted mean of ${M}_{l}5.0$. Our results for the tapered earthquake-size distribution of the Groningen catalogue and NW-region hint at a possible corner magnitude of ${M}_{l}3.4-3.5$ (Table 1; Fig. 2e). However, it is well known that this is an underestimate due to bias for small samples^21^. For the Groningen case, the underestimation will be of the order of 0.1–0.15 magnitude points^21^. Thus, we obtain a bias-corrected corner magnitude estimate of ${M}_{l}3.5-3.6$, which would correspond to an equivalent truncation of the earthquake-size distribution at about ${M}_{l}4.1$. If this would be considered an area-characteristic corner magnitude for the Groningen gas field, this would also explain the absence of any statistical information on the corner magnitude in most regions. After all, only a single event exceeding ${M}_{l}3.0$ (${M}_{l}3.1$ at Hellum on September 30, 2015) was observed outside the NW-region. Therefore, our results seem to suggest that the relative probability of the lower end of the current ${M}_{max}$ distribution^49^ should be increased, as the larger maximum possible magnitudes would not affect the probability of events with magnitudes as low as ${M}_{l}3.0-3.5$. Based on a variety of methods, Beirlant et al.^48^ concluded that the area-characteristic ${M}_{max}$ of the Groningen gas field should be in the range 3.61–3.8, with a 90% confidence upper bound of 3.85 to 4.5. Our results are consistent with the upper end of their estimates.

Finally, we note that in our analysis we reach the limits of the information that can be extracted from the data. The number of events available is very limited and thus the derived *b*-values prone to large uncertainties and bias. In the adopted systematic approach, we have taken great care to minimize the bias as much as possible, but cannot exclude that some bias due to the small sample sizes remains. Given the implications of the earthquake-size distribution on risk estimates, this emphasizes the great importance of early-stage, dedicated, high resolution monitoring of anthropogenic seismicity, to ensure large enough databases for accurate and robust statistical analyses.

## Conclusions

Our results show statistically significant spatial variations of the earthquake-size distribution in the induced seismicity sequence of Groningen. The probability of larger magnitude events in the NW-region is statistically significantly larger than in the southern and eastern parts of the gas field. These spatial variations will affect the regional probability of larger magnitude events and could be incorporated as a viable model alternative in the Groningen seismic hazard and risk assessment. We find no compelling, statistical evidence of a temporal variation.

Our analysis further shows that the occurrence probability of events with magnitudes exceeding ${M}_{l}3.0$ is lower than expected. Our results are consistent with the presence of an area-characteristic corner magnitude in the Groningen gas field around ${M}_{l}3.5$ or a maximum possible magnitude around ${M}_{l}4.1$. Our results imply that the current risk assessment models, which use the conditional ${M}_{max}$ distribution^49^, overestimate the probability of larger magnitude events (M ≥ 3.0) in the Groningen gas field and thus potentially the risk posed.

However, we emphasize that an upper bound should better not be inferred based on the limited Groningen catalogue alone. Especially for relatively small datasets, it is well known that these data assessments are prone to the presence of bias. This does not disqualify the assessments, but it is useful and vital to systematically study the sources of bias, as we have done in this paper.

The here presented, systematic and unbiased spatiotemporal analysis procedure does not only apply to the Groningen gas field, but is transferable to, and of the utmost relevance for, accurate risk analysis of any subsurface operation potentially causing induced seismicity, especially when the corresponding earthquake catalogues are relatively small. This potentially includes, but is certainly not limited to, geothermal energy, CO_2_ capture, sequestration and utilization, and hydrogen storage operations. All highly relevant in the context of the global energy transition.

Specific to the Groningen case, our conclusions can be considered a valuable addition to already existing evidence of either a tectonic or a reservoir limit on the maximum possible magnitude for the Groningen gas field, as was the case in the study by NAM^49^, especially considering the implications on hazard and risk estimates.

## Data Availability

Data used to produce the results of this study are freely available at the KNMI via https://www.knmi.nl/kennis-en-datacentrum/dataset/aardbevingscatalogus (Only available in Dutch).
